# The altered metabolites contributed by dysbiosis of gut microbiota are associated with microbial translocation and immune activation during HIV infection

**DOI:** 10.3389/fimmu.2022.1020822

**Published:** 2023-01-04

**Authors:** Yu Zhang, Zhiman Xie, Jie Zhou, Yanjun Li, Chuanyi Ning, Qisi Su, Li Ye, Sufang Ai, Jingzhen Lai, Peijiang Pan, Ningmei Liu, Yanyan Liao, Qijian Su, Zhuoxin Li, Hao Liang, Ping Cui, Jiegang Huang

**Affiliations:** ^1^ Guangxi Key Laboratory of AIDS Prevention and Treatment and Guangxi Universities Key Laboratory of Prevention and Control of Highly Prevalent Disease, Nanning, China; ^2^ School of Public Health, Guangxi Medical University, Nanning, China; ^3^ The Tenth Affiliated Hospital of Guangxi Medical University, Qinzhou, China; ^4^ Department of Infectious Diseases, The Fourth People's Hospital of Nanning, Nanning, China; ^5^ Life Science Institute, Guangxi Medical University, Nanning, China

**Keywords:** HIV, gut microbiota, metabolism, immune activation, microbial translocation

## Abstract

**Background:**

The immune activation caused by microbial translocation has been considered to be a major driver of HIV infection progression. The dysbiosis of gut microbiota has been demonstrated in HIV infection, but the interplay between gut microbiota and its metabolites in the pathogenesis of HIV is seldom reported.

**Methods:**

We conducted a case-controlled study including 41 AIDS patients, 39 pre-AIDS patients and 34 healthy controls. Both AIDS group and pre-AIDS group were divided according to clinical manifestations and CD4 + T cell count. We collected stool samples for 16S rDNA sequencing and untargeted metabolomics analysis, and examined immune activation and microbial translocation for blood samples.

**Results:**

The pre-AIDS and AIDS groups had higher levels of microbial translocation and immune activation. There were significant differences in gut microbiota and metabolites at different stages of HIV infection. Higher abundances of pathogenic bacteria or opportunistic pathogen, as well as lower abundances of butyrate-producing bacteria and bacteria with anti-inflammatory potential were associated with HIV severity. The metabolism of tryptophan was disordered after HIV infection. Lower level of anti-inflammatory metabolites and phosphonoacetate, and higher level of phenylethylamine and polyamines were observed in HIV infection. And microbial metabolic pathways related to altered metabolites differed. Moreover, disrupted metabolites contributed by altered microbiota were found to be correlated to microbial translocation and immune activation.

**Conclusions:**

Metabolites caused by dysbiosis of gut microbiota and related metabolic function are correlated to immune activation and microbial translocation, suggesting that the effect of microbiota on metabolites is related to intestinal barrier disruption in HIV infection.

## Introduction

Systemic immune activation is a feature of the HIV disease progression, characterized by persistent T cell activation and high levels of pro-inflammatory factors (1). It is now widely believed that chronic immune activation in HIV infection depends primarily on microbial translocation, a process by which gut microbes or their products move from the intestinal lumen into the portal vein and the circulatory system (1). Before and during acute HIV infection, intestinal CD4+ T lymphocytes are depleted, and intestinal mucosal epithelial integrity is destroyed (2–5). Recent research also reported that a decrease and dysfunction of mucosal-associated invariant T (MAIT) cells in HIV infection impair intestinal mucosal integrity and intestinal microbiota homeostasis (6). The defect of intestinal structure and immune function leads to gut microbiota dysbiosis and microbial translocation (1), followed by systemic immune activation, ultimately accelerating the depletion of CD4+ T cells and the progression to AIDS (1, 7).

Numerous observations have confirmed that the composition of gut microbiota is altered in HIV-infected individuals (8–16), and found gut microbiota plays a key role in microbial translocation and immune activation. Previous studies have shown that the reduction of butyrate-producing bacteria and *Bacteroides* have an impact on microbial translocation and immune activation (13, 14). *Lactobacillus* can reduce microbial translocation and delay disease progression by preserving Th17 cells that are able to maintain the integrity of the intestinal barrier (17). In addition to the alteration in gut microbiota, the microbial metabolic pathway also changed (11, 12). Genes involved in the pathogenic process, such as lipopolysaccharide (LPS) biosynthesis, microbial translocation and inflammatory pathways, were enriched in HIV-positive individuals (12), while metabolic pathways relevant to amino acid metabolism and anti-inflammatory processes were underexpressed (11). These genomic data focused on the effect of microbial taxa on HIV infection, while could not reveal the survival status and metabolic potential of gut microbiome (18).

Recently, changes in faecal metabolites have also been found in HIV-infected individuals (19–21). Metabolites in the gastrointestinal tract are mainly produced and modulated by the host microbiome, but are modestly affected by the host gene, which could reflect the composition and real activity of gut microbiota (22). Overall, microbial metabolic pathways are responsible for 95% of the metabolite generation or regulation (23). The metabolites enter the human body through the absorption of intestinal epithelium and play a role in the local or distal organs, affecting human health more directly than the microbial cells themselves (24). It has been showed that microbiome-associated metabolites have a profound effect on the mucosal and systemic immune function (25). Nevertheless, there has been little research on the role of gut metabolites in HIV infection, and studies regarding to the interplay between gut microbiota and metabolites in HIV disease progression are rare.

We integrated genomics and metabolomics in this study. 16S rDNA sequencing and non-targeted metabolomics profiling were performed on stool samples from subjects at different stages of HIV infection. We characterized the structure of the gut microbiome and metabolites during HIV infection, and investigated the relationship of gut metabolites with microbial translocation and immune activation, and the relationship between gut microbiota and metabolites. Our study aimed to explore metabolic potential of microbiome, and provide preliminary data for studying the role of the interplay between microbiome and metabolites in the pathogenesis of HIV.

## Methods

### Subject recruitment

Our research was conducted in Guangxi, one of the Chinese provinces with the largest number of people living with HIV (PLWH) (26). Untreated HIV/AIDS patients and uninfected individuals were recruited from the two hospitals in Nanning, the capital of Guangxi province in southern China (the First Affiliated Hospital of Guangxi Medical University and the Fourth People’s Hospital of Nanning), in 2018-2019. Referring to the Chinese guidelines for the diagnosis and treatment of HIV/AIDS (2021 edition) (27), ART-naïve untreated HIV-infected individuals were divided into the AIDS group and the pre-AIDS group according to CD4+ T lymphocyte counts and clinical manifestations. The groups were as follows: 1) AIDS patients: HIV-infected individuals with CD4+ T cell count ≤ 200 cells/μL, not receiving ART treatment, with or without AIDS indicator diseases; 2) Pre-AIDS patients: HIV-infected individuals with CD4+ T cell count >200 cells/μL, without ART treatment and no AIDS related disease; 3) Healthy controls: HIV-negative individuals. According to the Chinese AIDS diagnosis and treatment guidelines (2021 Edition), AIDS indicative disease include irregular fever above 38°C with unknown etiology lasting for more than 1 month, diarrhea with more than three stools per day lasting for more than 1 month, loss of more than 10% weight within 6months, recurrent oral fungal infection, recurrent infection of herpes simplex virus or herpes zoster virus, pneumocystis jirovecii pneumonia (PCP), recurrent bacterial pneumonia, active tuberculosis or nontuberculous mycobacteria (NTM) infection, invasive fungal infection, space-occupying lesions in central nervous system, dementia in young and middle-aged adults, active cytomegalovirus (CMV) infection, cerebral toxoplasmosis, talaromyces marneffei infection, recurrent septicemia, and Kaposi’s sarcoma or lymphoma. All participants were informed of the purpose of the study and the confidentiality of the investigation, and informed consent was signed by participants. The study was approved by the ethics committee of Guangxi Medical University (Approval number: 20180307-069).

### Blood and stool sample collection

Blood and stool samples were collected for each subject. A vacutainer tube containing EDTA (BD Vacutainer^®^) was used to collect 5 mL blood samples from each participant. Plasma and peripheral blood mononuclear cells (PBMCs) were isolated on the day of collection. The plasma was separated after centrifugation at 800 g for 10 min. Then, PBS was added to the blood cells at a ratio of 1:1, and PBMCs were isolated by centrifugation with Ficoll-plaque (GE Healthcare Buckinghamshire, UK) at 400 g for 45 min. Both plasma and PBMCs were frozen at -80°C before use. Stool samples were collected in stool collection tubes with stool DNA stabilizer (Stratec, Berlin, Germany) and stored at -80°C before use.

### Flow cytometry

PBMCs were thawed and then washed twice with PBS before being resuspended in PBS containing 0.5% foetal bovine serum (FBS) (Gibco, CA, USA). To block non-specific binding of the fluorescent antibody to receptors expressed on the cells, Human Fc Block was used before staining. After blocking, the cells were stained with antibodies at 4°C for 30 min and washed twice with PBS (Solarbio, Beijing, China). The antibodies used were: BB515 Mouse Anti-Human CD4, PerCP-Cy™5.5 Mouse Anti-Human CD8, APC Mouse Anti-Human CD38 and PE Mouse Anti-Human HLA-DR. Both Human Fc Block and antibodies were purchased from BD Bioscience (NJ, USA). T cells were identified by plotting the forward scatter area (FSC-A) against the side scatter area (SSC-A). The activated CD4+ and CD8+ T cells were defined by CD38+HLA-DR+ CD4+ T cells and CD38+HLA-DR+ CD8+ T cells, respectively. To analyse activated CD4+ and CD8+ T cells, the gates for CD4+ and CD8+ T cells were set on gated T cells, and the frequency of the activated T cells was measured by HLA-DR and CD38 expression gated from CD4+ and CD8+ T cells, respectively. Since the fluorescence spillover can affect the accuracy of gate setting, Fluorescence Minus One (FMO) control was used to more accurately set the boundary between negative and positive staining. Data were analyzed with CytExpert software and transferred into analysis with GraphPad Prism 8.0 (San Diego, CA, USA).

### Enzyme-linked immunosorbent assay (ELISA)

Plasma sCD14 and EndoCAb IgM were used to measure the level of microbial translocation in our study (28). Lipopolysaccharide (LPS) directly stimulates sCD14 production. When LPS enters the cycle, EndoCab IgM clears LPS by binding to it, and EndoCab IgM titres are reduced (28). High levels of sCD14 and low levels of EndoCab IgM are considered to be the increase of microbial translocation. sCD14 (R&D) and EndoCAb IgM (Hycult Biotech) in plasma were detected by an enzyme-linked immunosorbent assay. All experimental procedures were performed according to the manufacturer’s protocols.

### DNA extraction and 16S rDNA sequence data process

Total DNA was extracted by using the Fast DNA SPIN Extraction Kit (MP Biomedicals, CA, USA) according to the manufacturer’s instructions. The V3-V4 variable regions in 16S rRNA was amplified with primers 338F (5’-ACTCCTACGGGAGGCAGCAG-3’) and 806R (5’-GGACTACHVGGGTWCTAAT-3’) on a GeneAmp 9700 thermal cycler PCR system (Applied Biosystems, USA). PCR products were quantified and homogenized using a Picogreen dye fluorometer. The Illumina Miseq platform (Majorbio BioPharm Technology Co., Ltd., Shanghai, China) was used to sequence the PCR products of 300-500 bp.

To obtain effective sequences, the raw sequences were filtered by Fastp (version 0.19.6). Bases less than 20 at the tail of the reads were filtered, and a 50-bp window was set to filter the reads less than 50-bp. Reads with N-base were also removed. Paired reads were merged into a sequence with a minimum overlap length of 10-bp by using FLASH (version 1.2.11). The maximum mismatch ratio allowed in the overlapping region of the splicing sequence was 0.2, and unqualified sequences were filtered. Samples were distinguished according to the barcodes and primers, and the direction of the sequence was adjusted. The allowed mismatch number for the barcode was 0, and the maximum mismatch number for the primer was 2. UPARSE (version 7.0.1090, http://drive5.com/uparse/) was used to perform Operational taxonomic units (OTUs) clustering of non-repeating sequences (excluding singletons) with a cutoff of 97% similarity. Taxonomic analysis was conducted with RDP Algorithm (version 11.5, http://rdp.cme.msu.edu/) against the SILVA ribosomal RNA gene database (Release138 http://www.arb-silva.de/), with a confidence threshold of 70%. Similar approaches can be found in other studie (29).

### Metabolite extraction and LC-MS analysis

Metabolites were extracted from a stool sample. A 100 mg faecal sample was transferred into 2 mL centrifuge tubes, and 500μL ddH_2_O (4°C) and 1ml methanol (pre-cooled at -20°C) were added successively to vortex. The centrifugal tube was placed in the ultrasonic machine for 10 min at room temperature, and then placed on ice for 30 min before centrifugation at 14000 rpm at 4°C for 10 min. After centrifugation, 1200 μL supernatant was extracted into a new centrifuge tube and then concentrated. The concentrated supernatant was dissolved with 400 μL 4 ppm 2-chlorophenylalanine (methanol configuration), and filtered by 0.22-μm membrane to obtain the sample to be tested. 20 µL of each sample was taken and blended as a quality control (QC) sample for monitoring of deviations in the analytical results. The rest of the sample was used for untargeted metabolite detection with Liquid Chromatograph-Mass Spectrometry (LC-MS) methods. Chromatographic separation was accomplished by Thermo Ultimate 3000 system equipped with an ACQUITY UPLC^®^ HSS T3 (2.1 × 150 mm × 1.8 mm; Waters, Milford, MA,USA).) column maintained at 40°C. Thermo Q Exactive Focusmass spectrometer (ThermoFisher Scientific, USA) with both positive and negative ESI models was used for detection of metabolites.

The raw data were converted to mzXML format by Proteowizard software (v3. 0.8789), and the R (v3.3.2) XCMS package was used for peak identification, peak filtration and peak alignment. The data matrix of different characteristic peaks with mass-to-nuclear ratio (m/z), retention time (RT) and intensity was obtained and organized into a table. To enable data from different magnitudes to be compared, the data were normalized by a batch of peak areas. Characteristic peaks with a relative standard deviation (RSD) greater than 30% of the QC sample were excluded. The identification of metabolites was first confirmed based on the exact molecular weight (molecular weight error <20 ppm), and then the MS/MS fragmentation mode was used to further confirm and annotate metabolites against the Human Metabolome Database (HMDB) (http://www.hmdb.ca), LipidMaps (http://www.lipidmaps.org), Metlin (http://metlin.scripps.edu), massbank (http://www.massbank.jp/), mzclound (https://www.mzcloud.org) and database built by BioNovoGene Co., Ltd (30) (Suzhou, China).

### Bioinformatics analysis

For sequence data analysis, Major Bio Cloud Platform (https://cloud.majorbio.com/) was used to perform (29). Principal co-ordinates analysis (PCoA) calculated by Bray-Curtis was carried out to analyse the difference in gut microbiota, and permutational multivariate ANOVA (PERMANOVA) was performed to test the significance. Partial Least Squares Discriminant Analysis (PLS-DA) was performed to visually present the clusters of microbiota at different stages of HIV infection. The Kruskal-Wallis H test was used to compare differences of microbiota at genus level or species level between different groups. The Kyoto Encyclopedia of Genes and Genomes (KEGG) pathway level 3 was analyzed with PICRUSt2 for microbial function predictions (31), and ANOVA was used to compare distinction of the KEGG pathway. The Benjamini-Hochberg procedure was used to correct, and FDR *P*<0.10 was considered significant.

For metabolomics analysis, R language Ropls package was used for Orthogonal Projections to Latent Structures Discriminant Analysis (OPLS-DA) to reveal differences in the metabolic compositions, and the importance in projection (VIP) was obtained from the OPLS-DA. One-way ANOVA was used to analyse differences of each metabolites. Metabolites with VIP>1.0 and *P*<0.05 were identified as differential metabolites.

### Statistical analysis

Participant characteristics were summarized and compared with frequencies (%) for categorical variables and means (standard deviation) or median (interquartile range [IQR]) for continuous variables by using SPSS Statistics 20.0 (Chicago, IL, USA). The comparison of gender or race at different stages of HIV infection was compared by Chi-square test, and the one-way ANOVA was applied to compare difference in age. The CD4 count, markers of microbial translocation (plasma sCD14 and EndoCAb IgM) and immune activation (proportion of activated CD4+ and CD8+ T cells) in different groups were compared with Kruskal-Wallis rank-sum test. The data were graphically plotted using GraphPad Prism 8.0 to show the difference in markers of microbial translocation and immune activation, gut microbiota, microbial function and metabolites. Spearman correlation analysis was used to study the correlation between gut microbiota, differential metabolites and disease indicators. The correlation between differential metabolites and disease indicators was displayed by heat map using GraphPad Prism 8.0, and heat map of correlation between microbiota and differential metabolites were drawn by Major Bio Cloud Platform. According to the matrix generated by correlation analysis, the interplay between microbiota, metabolites and disease indicators were drawn with R software 4.1.3 (R Foundation for Statistical Computing, Vienna, Austria).

## Results

### Participant characteristics

A total of 114 participants were recruited, including 41 AIDS patients, 39 pre-AIDS patients and 34 healthy controls. As shown in **Table 1**, except for CD4+ T cell counts, there were no significant differences in age, gender, or ethnicity among the three groups (all *P*>0.05).

**Table 1:** Baseline characteristics and clinical parameters of study participants.

	AIDS (n=41, %)	Pre-AIDS (n=39, %)	Healthy control (n=34, %)	*F/χ* ^2^	*P* value
Age				1.083	0.342
Mean ± SD	45.7 ± 14.3	41.6 ± 14.1	41.8 ± 13.4		
Gender				0.626	0.731
Male	30 (73.2)	27 (69.2)	22 (64.7)		
Female	11 (26.8)	12 (30.8)	12 (35.3)		
Race				4.367	0.113
Han	17 (41.5)	18 (46.2)	22 (64.7)		
Others	24 (58.5)	21 (53.8)	12 (35.3)		
CD4+ T cell count (cells/μl)				-	-
Median (IQR)	31.0 (10.5, 76.0)	360.0 (291.0, 420.0)	Not available	-	-

### High levels of microbial translocation and immune activation were related to HIV severity

**Figure 1:**
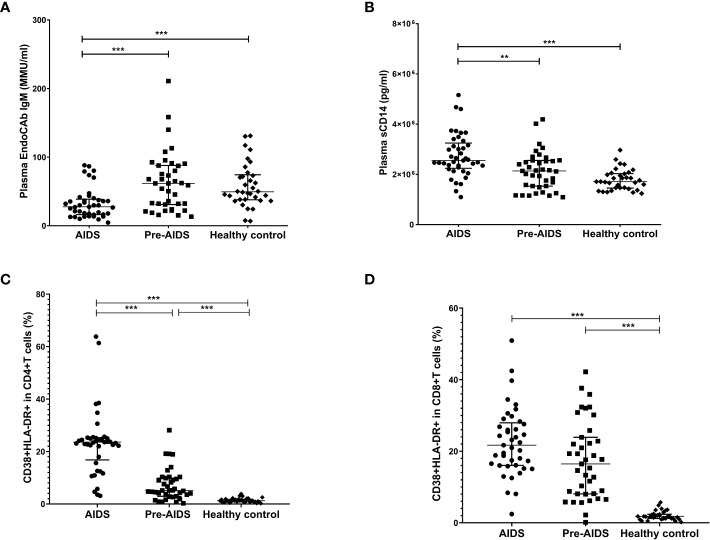
Markers of microbial translocation and immune activation in the AIDS group, pre-AIDS group and the healthy control group. Compared with the pre-AIDS and healthy control groups, EndoCAb IgM levels were significantly lower **(A)**, and plasma sCD14 levels were significantly higher **(B)**. The AIDS and pre-AIDS groups had a significantly higher proportion of activated CD4+ T cells **(C)** and CD8+ T cells **(D)** than the healthy control group. **: 0.001 ≤ *P* < 0.010; ***: *P* < 0.001.

The AIDS group had lower levels of plasma EndoCAb IgM when compared to the pre-AIDS group and the healthy control group (**Figure 1A**), while had higher levels of plasma sCD14 (*P*<0.001) (**Figure 1B**). Compared with the healthy control group, the proportion of activated CD4+ T cells and CD8+ T cells were significantly higher in the AIDS and pre-AIDS groups, and the proportion of activated CD4+ T cells increased significantly with the severity of the disease (**Figures 1C, D**) (*P*<0.01). Example of intracellular activated CD4+ and CD8+ T cells analyzed with CytExpert software are shown in **Figure S1**. Details of these are summarized in **Table S1**.

### Gut microbiota was significantly different at different stages of HIV infection

**Figure 2:**
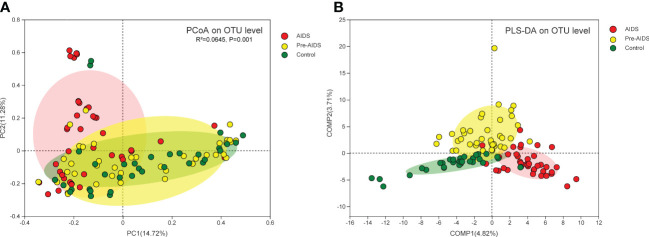
Composition of gut microbiota at different stages of HIV infection. **(A)** PCoA of Bray-Curtis dissimilarity of microbiota revealed that gut microbiota was separated by different stages of HIV infection, and a statistically significant difference was observed in the PERMANOVAR (*P*=0.001); **(B)** PLS-DA showed that gut microbiota at different stages of HIV infection clustered into three distinct clusters. Each dot refers to a sample. Red dot, yellow dot and green dot represent AIDS group, pre-AIDS group and healthy control group respectively.

As shown in **Figure 2A**, PCoA with Bray-Curtis dissimilarity and PERMANOVA revealed a significant difference in the microbiota composition at different stages of HIV infection (*P*=0.001), and PLS-DA showed that gut microbiota at different stages of HIV infection were clearly separated (**Figure 2B**).

**Figure 3:**
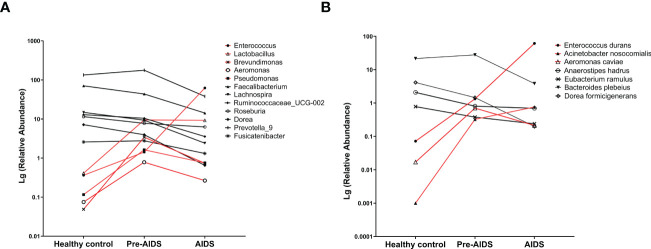
Gut microbiota at different stages of HIV infection. Difference of bacterial genera **(A)** and bacterial species **(B)** at different stages of HIV infection. Compared with healthy controls, higher abundance bacteria (red lines) and lower abundance bacteria (black lines) in AIDS and pre-AIDS patients were observed.

To determine which bacteria had changed at different stages of HIV infection, bacteria were analyzed at genus level or species level. At the genus level, AIDS and pre-AIDS groups had higher relative abundance of *Lactobacillus*, pathogenic bacteria and opportunistic pathogens, such as *Enterococcus* (32), *Brevundimonas* (33), *Aeromonas* (34, 35) and *Pseudomona*s (36). The abundance of *Enterococcus* and *Lactobacillu*s shown an upward trend with the severity of HIV disease, while that of butyrate-producing bacteria, such as *Faecalibacterium*, *Lachnospira*, *Ruminococcaceae_UCG-002*, *Roseburia* and *Dorea* (37, 38), shown a downward trend. The relative abundance of *Prevotella_9* (39) and *Fusicatenibacter* (40) in AIDS group, which were found to be associated with alleviating inflammation, were lower than pre-AIDS and healthy control group (FDR *P*<0.1). (**Figure 3A**).

At the species level, *Enterococcus durans (* 32, 41)*, Acinetobacter nosocomialis* (42, 43), and *Aeromonas caviae* (35) which are pathogenic to humans, were enriched in the pre-AIDS and AIDS groups, and *Enterococcus durans* and *Acinetobacter nosocomialis* also gradually enriched in patients with the severity of HIV disease. The relative abundance of *Anaerostipes hadrus* and *Eubacterium ramulus*, which are also butyrate-producing bacteria (44, 45), shown a downward trend with the severity of HIV disease. The relative abundance of *Bacteroides plebeius*, which was found to be associated with alleviating inflammation in another study (41), had a downward trend with the severity of HIV disease. (FDR *P*<0.1) (**Figure 3B**). Details can be seen in **Table S2**.

### Gut metabolites at different stages of HIV infection

**Figure 4:**
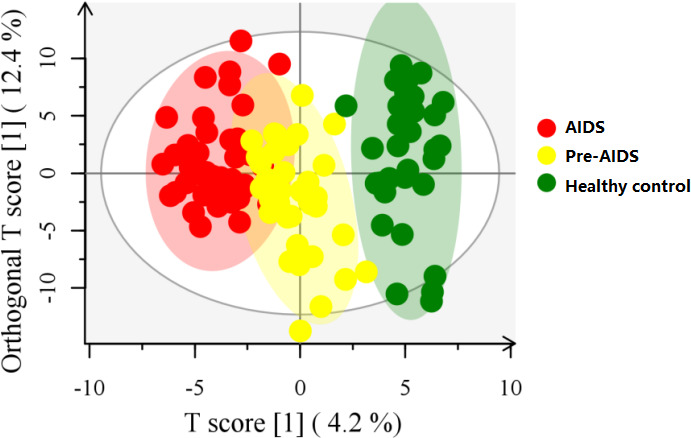
OPLS-DA score plot at different stages of HIV infection. Gut metabolites was clearly separated at different stages of HIV infection.

**Figure 5:**
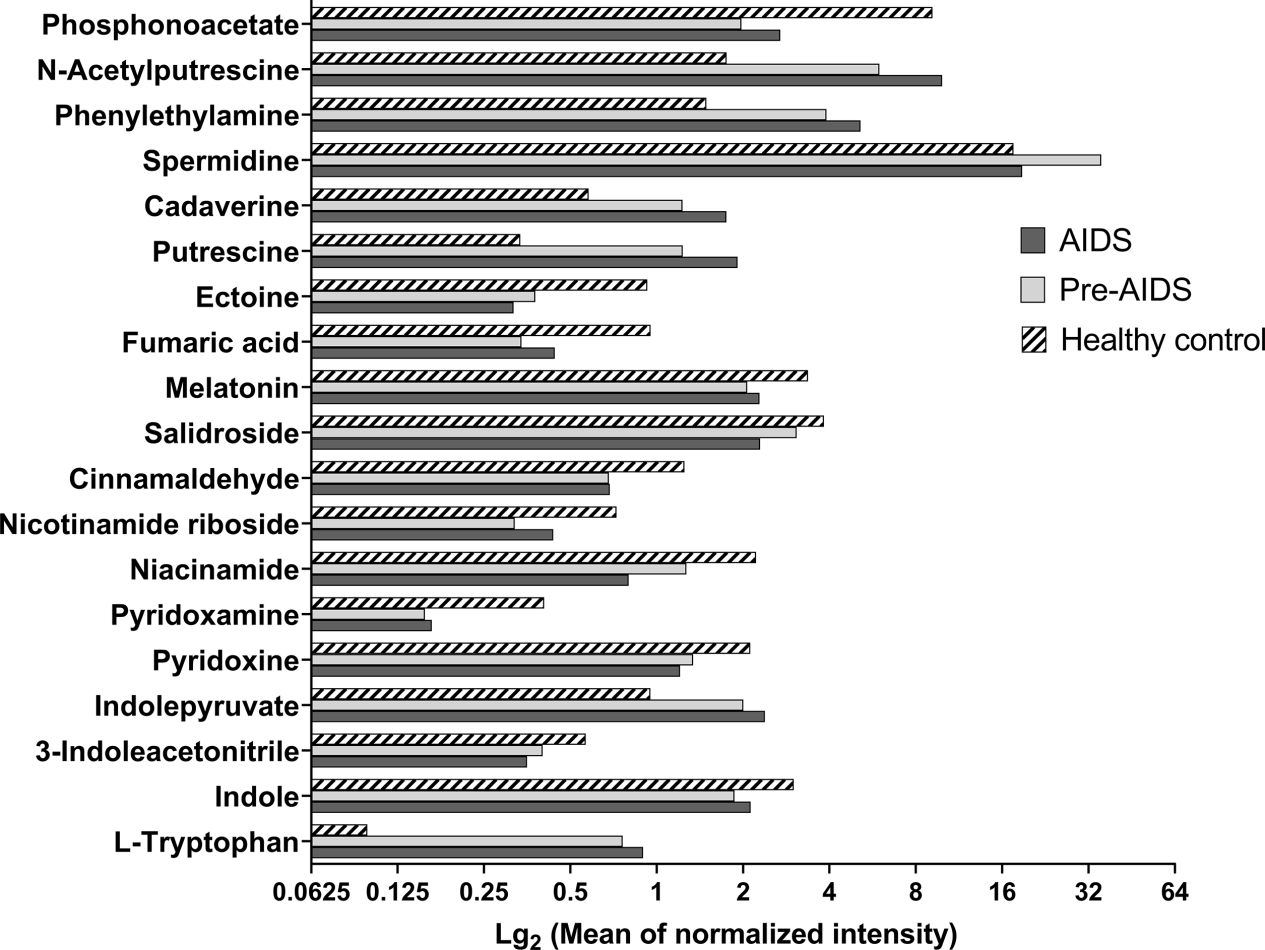
Differential metabolites at different stages of HIV infection. Metabolites with VIP>1.0 and P<0.05 were considered as differential metabolites. Compared with healthy controls, AIDS and pre-AIDS groups had lower levels of phosphonoacetate, pyridoxine, pyridoxamine, niacinamide, nicotinamide riboside, indole, 3-indoleacetonitrile, ectoine, salidroside, cinnamaldehyde, melatonin, and fumaric acid. And higher levels of putrescine, cadaverine, N-acetylputrescine, phenylethylamine, spermidine, indolepyruvate, and L-tryptophan were observed in AIDS and pre-AIDS groups.

A total of 354 gut metabolites were identified. As shown in **Figure 4**, a clear separation in metabolites was observed at different stages of HIV infection (R^2^Y=80.7%, Q^2^=48.9%). Differential metabolites are shown in **Figure 5** (*P*<0.05 and VIP>1.0). In terms of tryptophan metabolism, levels of indolepyruvate and L-tryptophan shown an upward trend with the severity of HIV disease, while indole, 3-indoleacetonitrile levels were lower in pre-AIDS and AIDS group compared to healthy control group. At the same time, AIDS and pre-AIDS groups had lower levels of phosphonoacetate and anti-inflammatory metabolites, such as vitamin B3 (niacinamide and nicotinamide riboside) (46, 47), vitamin B6 [pyridoxine (48) and pyridoxamine (49)], ectoine (50), cinnamaldehyde (51), salidroside (52, 53), melatonin (54, 55) and fumaric acid (56), among which niacinamide, pyridoxine, ectoine, cinnamaldehyde and salidroside exhibited a decreasing trend with the severity of disease. Furthermore, except for spermidine, levels of other biogenic amines had an upward trend with the disease severity, including polyamines (putrescine, cadaverine, N-acetylputrescine) and phenylethylamine. There was no significant difference in butyric acid between the three groups (**Figure S2**), although we observed a low abundance of butyrate-producing bacteria in AIDS and pre-AIDS patients.

### Altered microbiota were correlated with metabolites involved in microbial translocation and immune activation.

**Figure 6:**
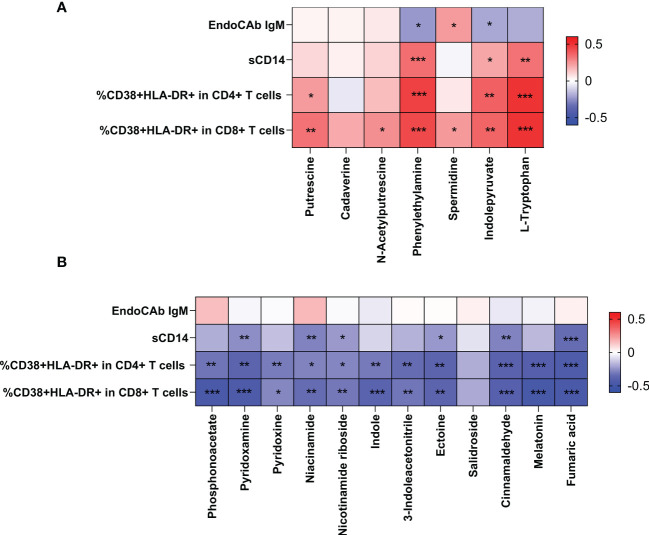
Correlation of the differential metabolites with immune activation and microbial translocation. The positive Spearman rank correlation coefficients are denoted by red squares, and negative correlation coefficients by blue squares. The metabolites, exhibited higher levels in HIV-positive individuals, were positively correlated with microbial translocation and immune activation **(A)**. In contrast, metabolites, exhibited lower levels in HIV-positive individuals, were negatively correlated with immune activation and microbial translocation **(B)**. *: 0.010 ≤ P < 0.050; **: 0.001 ≤ P < 0.010; ***: P < 0.001.

To study the role of metabolites during HIV infection, we first analyzed the relationship between the differential metabolites and indicators of microbial translocation. As shown in **Figure 6**, higher plasma sCD14 and higher proportion of activated CD4+ T cells and CD8+ T cells were positively correlated with higher levels of metabolites in both AIDS and pre-AIDS patients, such as L-tryptophan (**Figure 6A**), while negatively correlated with the lower levels of metabolites, like pyridoxamine, niacinamide, nicotinamide riboside, ectoin, cinnamaldehyde, and fumaric acid (**Figure 6B**) (*P*<0.05). Besides, higher proportion of activated CD4+ T cells and CD8+T cells were negatively correlated with phosphonoacetate, and positively correlated with putrescine.

**Figure 7:**
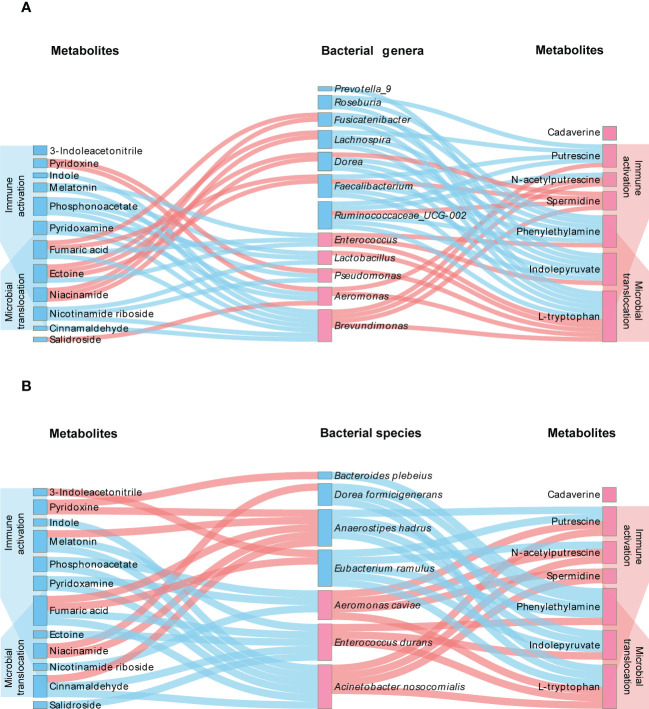
Interplay between differential microbiota, metabolites and indicators of disease severity. The genera **(A)** or species **(B)** of bacteria with higher and lower abundance in HIV-infected patients are distinguished by pink labels and blue labels, respectively, in comparison with healthy controls. The metabolites with pink and blue labels represent higher and lower levels of metabolites in HIV-infected patients, respectively. The pink line refers to a positive correlation between microbiota and metabolites, and the blue pink refers to a negative correlation (*P*<0.05). On both sides of the figure, it can be observed that metabolites are significantly negatively (blue) and positively (pink) associated with immune activation and microbial translocation (*P*<0.05).

We then analyzed the relationship between microbiota and differential metabolites. As shown in **Figure 7**, significant correlations of altered microbiota with metabolites and correlations of metabolites with microbial translocation and immune activation were identified. Among the altered microbiota, *Enterococcus*, *Enterococcus durans* and *Lactobacillus*, displaying notably higher abundance in HIV-positive individuals, were positively correlated with L-tryptophan. And *Enterococcus* and *Enterococcus durans* were positively correlated with phenylethylamine. We also found that bacteria with anti-inflammatory potential were positively correlated with anti-inflammatory metabolites. *Fusicatenibacter* were positively correlated with niacinamide and fumaric acid, and *Bacteroides plebeius* were positively correlated with pyridoxine. The results of relationship between gut microbiota and metabolites are shown in **Tables S3**-**S6** and **Figures S3**-**S6**.

### Microbial metabolic profiling

**Figure 8:**
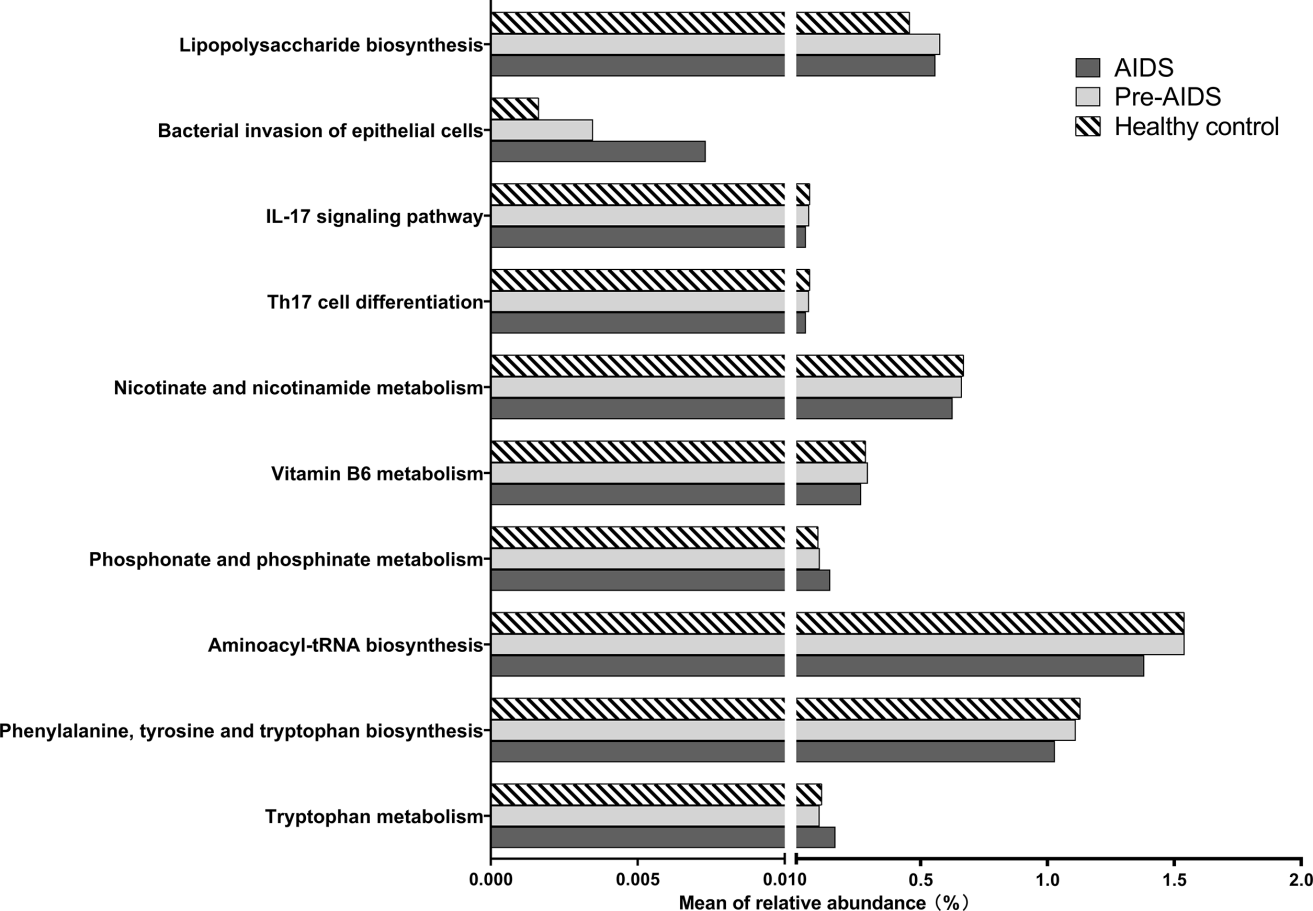
Alteration of KEGG pathways at different stages of HIV infection. In the context of HIV infection, the “bacterial invasion of epithelial cells”, “phosphonate and phosphate metabolism”, and “tryptophan metabolism” and pathways were up-regulated in AIDS group, while the “vitamin B6 metabolism” pathways were down-regulated (FDR P<0.1). The “IL-17 signalling pathway”, “Th17 cell differentiation”, “nicotinate and nicotinamide metabolism”, “aminoacyl-tRNA biosynthesis”, and “phenylalanine, tyrosine and tryptophan biosynthesis” showed a downward trend with the severity of the disease (FDR P<0.1).

In order to study the gut microbiome’s functional profiles, we analyzed the alteration in the KEGG pathway with 16S rDNA sequencing data. As shown in **Figure 8**, distinction in microbiota metabolic functions were observed at different stages of HIV infection. In the context of HIV infection, the *“IL-17 signalling pathway”* and *“Th17 cell differentiation”* showed a downward trend with the severity of the disease. Among the pathways related to tryptophan, the *“tryptophan metabolism”* pathway were up-regulated in AIDS group, while the *“aminoacyl-tRNA biosynthesis”* and *“phenylalanine, tyrosine and tryptophan biosynthesis”* pathways were down-regulated. AIDS and pre-AIDS patients had lower levels of vitamin B6 and vitamin B3, and the down-regulation of *“vitamin B6 metabolism”* and *“nicotinate and nicotinamide metabolism”* was observed in the AIDS group. *“phosphonate and phosphinate metabolism”* was up-regulated in the AIDS group, and lower level of phosphonoacetate was found in HIV infection. In addition, *“lipopolysaccharide biosynthesis”* and *“bacterial invasion of epithelial cells”* were up-regulated in HIV-positive individuals (FDR *P*<0.1).

## Discussion

Intestinal microbiota, intestinal epithelium and mucosal immunity together constitute the gut barrier (57). Evidence have confirmed that the destruction of the gut barrier can increase microbial translocation and maintain a high level of immune activation, thereby defects in the gut are thought to contribute to the progression of HIV infection (58). Here, we found higher level of microbial translocation and immune activation were associated with disease severity, as reported in prior studies (59). By performing 16S rRNA gene sequencing and untargeted metabolomics, we not only observed an alteration in gut microbiota and metabolites at different stages of HIV infection, but have also presented that gut microbiota is associated with metabolites involved in immune activation and microbial translocation for the first time.

Dysbiosis of microbiota composition is believed to alter the functional capacities of the microbiota. By analysis of functional profiles of microbiota, we observed an alteration in microbial immune pathway. Th17 cells are essential in maintaining mucosal barrier function by producing IL-17 and IL-22 to controll extracellular bacteria and enhance epithelial regeneration. Lower sigmoid Th17 cells in HIV-infected individuals is correlated with increased microbial translocation (60), and the loss of the Th17 cells in the intestinal tract promoted the progression of HIV-1 infection (61, 62). Th17 differentiation and immune response are affected by gut microbes (57). In our study, down-regulated genes of the *“IL-17 signalling pathway”* and *“Th17 cell differentiation”* were associated with severity of the disease, which has seldom been reported. The up-regulated *“lipopolysaccharide biosynthesis”* pathway in HIV infection was reported to be associated with microbial translocation (12), and enrichment of *“bacterial invasion of epithelial cells”* pathway damaged intestinal epithelium (63). Variation in these microbial function may impair the gut barrier. The interplay between gut microbiota and plasma metabolites in HIV-infected individuals has been reported (9), but few studies have elucidated the link between microbiota and metabolites in the gut. In our study, we found dysbiosis of microbiota and related metabolic function were associated with altered metabolites, indicating that changes of microbiota may affect the generation of metabolites. More importantly, these altered metabolites show association with microbial translocation and immune activation. Our results suggested that, except for the direct effect of bacteria, the microbiota may implicate in gut barrier injury by regulating metabolites in progression of HIV infection.

In this present study, we observed higher abundance of pathogenic bacteria and opportunistic pathogens in HIV-infected patients, such as *Enterococcus* (*Enterococcus durans*) (32, 41), *Brevundimonas* (33), *Aeromonas* (*Aeromonas caviae*) (34, 35), *Pseudomona*s (36) and *Acinetobacter nosocomialis* (42, 43). Under normal conditions, the specific composition of gut microbiota can maintain the intestinal barrier, and prevent the colonization of pathogenic bacteria by competing for nutrients or by inducing the production of inhibitory substances (64). Dysbiosis of the microbiome may impair resistance to colonization of pathogenic bacteria and opportunistic pathogens. More importantly, notably higher abundance of *Enterococcus, Enterococcus durans* and *Lactobacillus* was found in AIDS group, as well as an upward trend with the severity of HIV disease, conform with previous researches (65, 66). These bacteria were associated with metabolites involved in microbial translocation and immune activation in our study. However, the role of these bacteria in the pathogenesis of HIV infection and AIDS are still undefined.

With the gradual enrichment of *Lactobacillus* and *Enterococcus durans* in HIV infection, L-tryptophan levels had an upward trend, and alteration in microbial pathways of L-tryptophan synthesis and catabolism was observed. Observations have demonstrated changes of tryptophan and its metabolites in the plasma of HIV-infected individuals is associated with impaired mucosal immunity and microbial translocation (1, 67). However, tryptophan metabolism in gut has rarely been reported. The *Lactobacillus* and *Enterococcus durans* are able to synthesize tryptophan (68), in agreement with our findings that they were associated with L-tryptophan. About 95% tryptophan in the intestine is absorbed and degraded by indoleamine 2,3-dioxygenase 1 (IDO1) in immune cells and intestinal epithelial cells (IECs) into tryptophan catabolites (69), which inhibit the differentiation of TH 17 cells and reduce IL-17 and IL-22 produced by natural killer (NK) cells, potentiating microbial translocation (1). We found higher L-tryptophan levels were correlated with microbial translocation and immune activation, which may be related to the increased production of tryptophan catabolites *via* IDO1 that promote disruption of gut barrier. Indole and its derivatives are directly converted from tryptophan by gut microbes, and involved in a series of complex immune responses and immune cell differentiation in the intestine (69–71). Relationship of indole and its derivatives with microbial translocation and immune speculated that a disturbed tryptophan metabolism may impaired mucosal immunity. Meanwhile, *Enterococcus* and *Enterococcus durans* were observed to be correlated to phenylethylamine, which demonstrated an upward trend with severity of disease. Phenylethylamine is produced by a variety of *Enterococcus* species including *Enterococcus durans (* 72). In our study, higher phenylethylamine levels in HIV-positive individuals were related to microbial translocation and immune activation, presumably due to shedding of intestinal epithelial cells caused by its high concentrations in gut (73). The *Enterococcus* species may promote microbial translocation by generating higher levels of phenylethylamine, which further activates immunity. From the above results, it can be inferred that, enrichment of *Enterococcus, Enterococcus durans* and *Lactobacillus* is likely to have an impact on gut barrier by means of producing more metabolites affecting barrier function.

Altered microbiota were also correlated to low anti-inflammatory metabolites levels, such as vitamin B3 (46, 47), vitamin B6 (48, 49), ectoine (50), cinnamaldehyde (51), fumaric acid (56), ect. Among the altered microbiota, we observed notably lower abundance of bacteria relevant to the alleviation of inflammation in AIDS group, like *Prevotella_9* (39), *Fusicatenibacter (* 40) and *Bacteroides plebeius (* 41), in line with the increase of bacteria with pro-inflammatory potential previously found in HIV-infected people (21). It has been reported that reduced *Fusicatenibacter* was associated with intestinal inflammation (40), and *Bacteroides plebeius* was associated with remission of Crohn’s disease (CD) (41). Although the specific mechanism of how these bacteria perform anti-inflammatory effects in intestine remains unclear, we found they were associated with anti-inflammatory metabolites, probably mediated *via* regulation of metabolites. Reduction of anti-inflammatory metabolites in the gut causes inflammation (74). And high level of local and systemic inflammation cause enterocyte loss, increasing gut permeability in HIV infection (1). These metabolites were found to be associated with higher levels of microbial translocation and immune activation, indicating that the low levels of anti-inflammatory metabolites attributed to altered microbiota may be related to intestinal epithelial damage. Particularly, the levels of pyridoxine and niacinamide were correlated to changes of microbiota and microbial function involve in B vitamins catabolism, and presented a downward trend with the severity of disease. Pyridoxamine protects epithelial barrier by reducing the production of advanced glycation end products (AGEs) (75). Niacinamide is able to promote the secretion of antimicrobial peptides (AMPs) in human gut (46, 76) and maintains the intestinal epithelium barrier (77). A deficiency of B vitamins caused by dysbiosis of the microbes may influence the gut immunity, leading to furthering immune activation.

Polyamines that participate in replication of retroviruses (78–80), had an upward trend with the severity of disease, and were correlated to microbial translocation and immune activation. Nevertheless, low levels of phosphoonoacetate with the ability to inhibit HIV reverse transcription (81, 82) in HIV-positive individuals were negatively correlated with immune activation. HIV replication can drive immune activation by activating lymphocytes and macrophages (83, 84). High levels of polyamines and low levels of phosphoonoacetate may facilitate immune activation by promoting replication of virus. Previous studies have shown that no significant associations of viral load with β-diversity (9)and α-diversity (85, 86), indicating that changes in gut microbiota may be independent of viral load. It has been shown that in ART-naïve untreated (VU) individuals with high HIV RNA levels, their metabolic profiles were similar to those of immunological ART non-responders (INR) with extremely low level of HIV RNA, it may be influenced by other confounding factors (20). Viral load for these ART-naïve untreated individuals were not available in this study, but we observed altered microbiota were related to metabolites involved in HIV replication. The relationship between plasma viral load and metabolites affecting viral replication in HIV patients needs further study. Furthermore, the abundance of butyrate-producing bacteria showed a down-ward trend with the severity of the disease, and were negatively associated with metabolites involved in microbial translocation and immune activation, in accordance with other study (14). However, butyric acid showed no significant decrease in HIV infection, which may be affected by the survival status of gut microbiota.

Although we found interplay between gut microbiome and metabolites were correlated to immune activation and microbial translocation at different stages of HIV infection, some limitations of our study should be noted. Due to the nature of case-controlled study, this study is unable to demonstrate causal relationship between the gut microbiome, metabolites and HIV infection. Further experiments are required to explore how the gut microbiota affects metabolites and the specific role of gut metabolites in immune activation and microbial translocation. According to the sequencing data, this study has found that changes in metabolic pathways are related to altered metabolites. However, the metabolic pathway of bacteria is complicated, and the exact effect of metabolic pathway on products needs further experimental research to determine. Furthermore, 16S rDNA sequencing data and metabolome data in this study was corrected by FDR to reduce the false positive rate, but the use of antibiotics for some AIDS patients with opportunistic infections or other AIDS-indicative diseases, may affect the results.

In summary, our study found that gut microbiota and their metabolic function were relate to altered metabolites, and metabolites contributed by dysbiosis of gut microbiota were involved in microbial translocation and immune activation. Our data proposed the potential microbiota-metabolite-host interaction at different stages of HIV infection, providing new insight regarding the links between metabolites affected by microbiota and gut barrier disruption in HIV infection.

## Data availability statement

The datasets presented in this study can be found in online repositories. The names of the repository/repositories and accession number(s) can be found below: https://www.ncbi.nlm.nih.gov/, PRJNA810567.

## Ethics statement

The studies involving human participants were reviewed and approved by the ethics committee of Guangxi Medical University Approval number: 20200095. The patients/participants provided their written informed consent to participate in this study.

## Author contributions

JH, HL, PC, and ZX designed the study. YJL, QSS, SA, and NL participated in sample collection. CN, LY, JL, PP, YYL, QJS, and ZL contributed to data analysis. YZ and JZ participated in conducting experiments, interpreting the results and preparing the report for publication. All authors revised the manuscripts critically and approved the final version for publication. All authors revised the manuscripts critically and approved the final version for publication.

## Supplementary material

Supplementary Figure 1 The activated CD4+ and CD8+ T cells at the different stages of HIV infection. Example of intracellular activated CD4+ and CD8+ T cells from PBMCs of AIDS patients **(A-D)**, pre-AIDS patients **(E-H)** and healthy controls **(I-L)**.

Supplementary Figure 2 Butyric acid at different stages of HIV infection.

Supplementary Figure 3 The association between bacterial genera and metabolites positively correlated with immune activation and microbial translocation.

Supplementary Figure 4 The association between bacterial species and metabolites positively correlated with immune activation and microbial translocation.

Supplementary Figure 5 The association between bacterial genera and metabolites negatively correlated with immune activation and microbial translocation.

Supplementary Figure 6 The association between bacterial species and metabolites negatively correlated with immune activation and microbial translocation.
