# Supplementary figures and images for: The altered metabolites contributed by dysbiosis of gut microbiota are associated with microbial translocation and immune activation during HIV infection
Source: Front Immunol. 2023 Jan 4;13:1020822. doi: 10.3389/fimmu.2022.1020822 (PMC9845923; doi:10.3389/fimmu.2022.1020822)

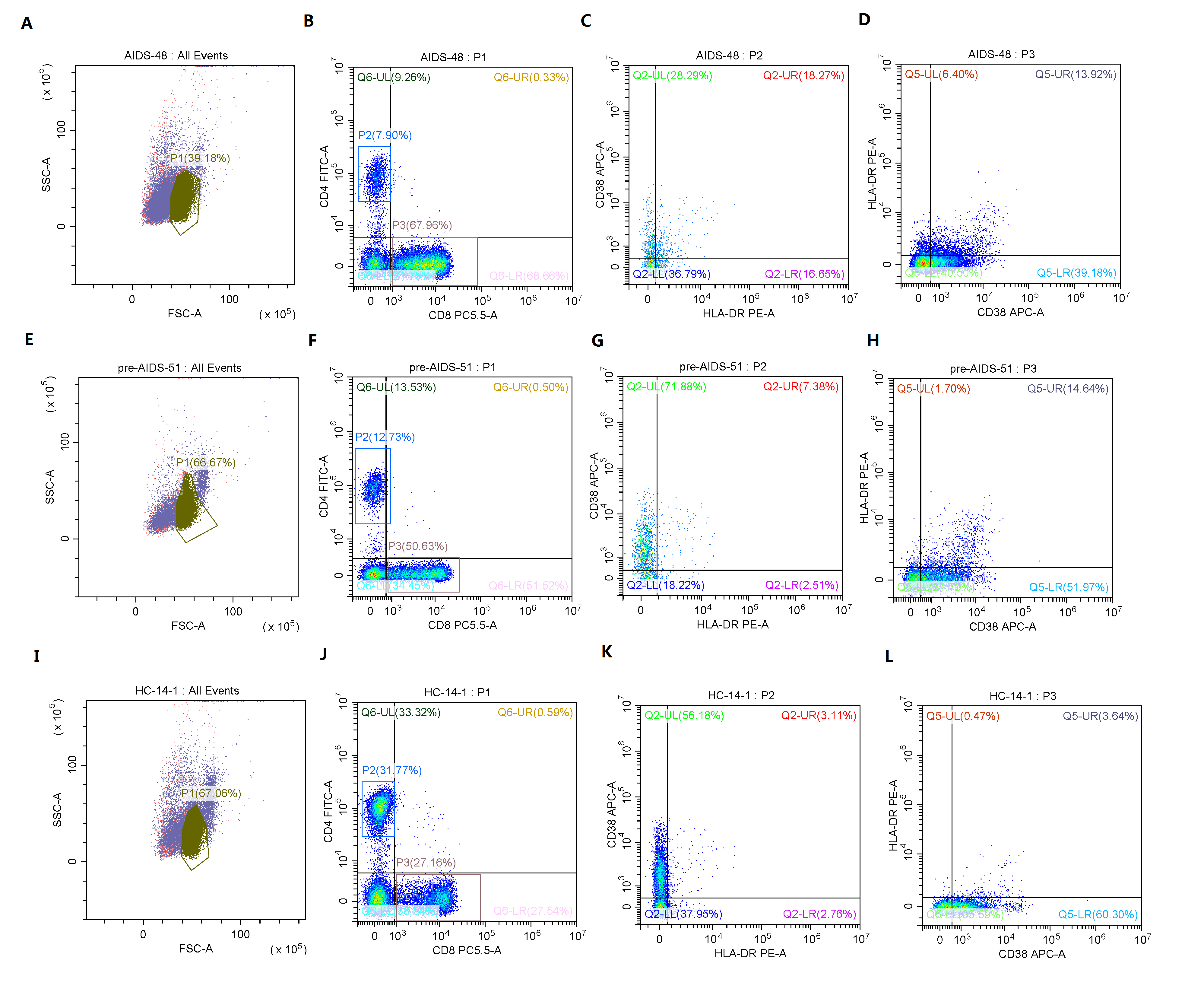

Supplement: Supplementary Figure 1 — The activated CD4+ and CD8+ T cells at the different stages of HIV infection. Example of intracellular activated CD4+ and CD8+ T cells from PBMCs of AIDS patients (A-D), pre-AIDS patients (E-H) and healthy controls (I-L). [file Image_1.tif]

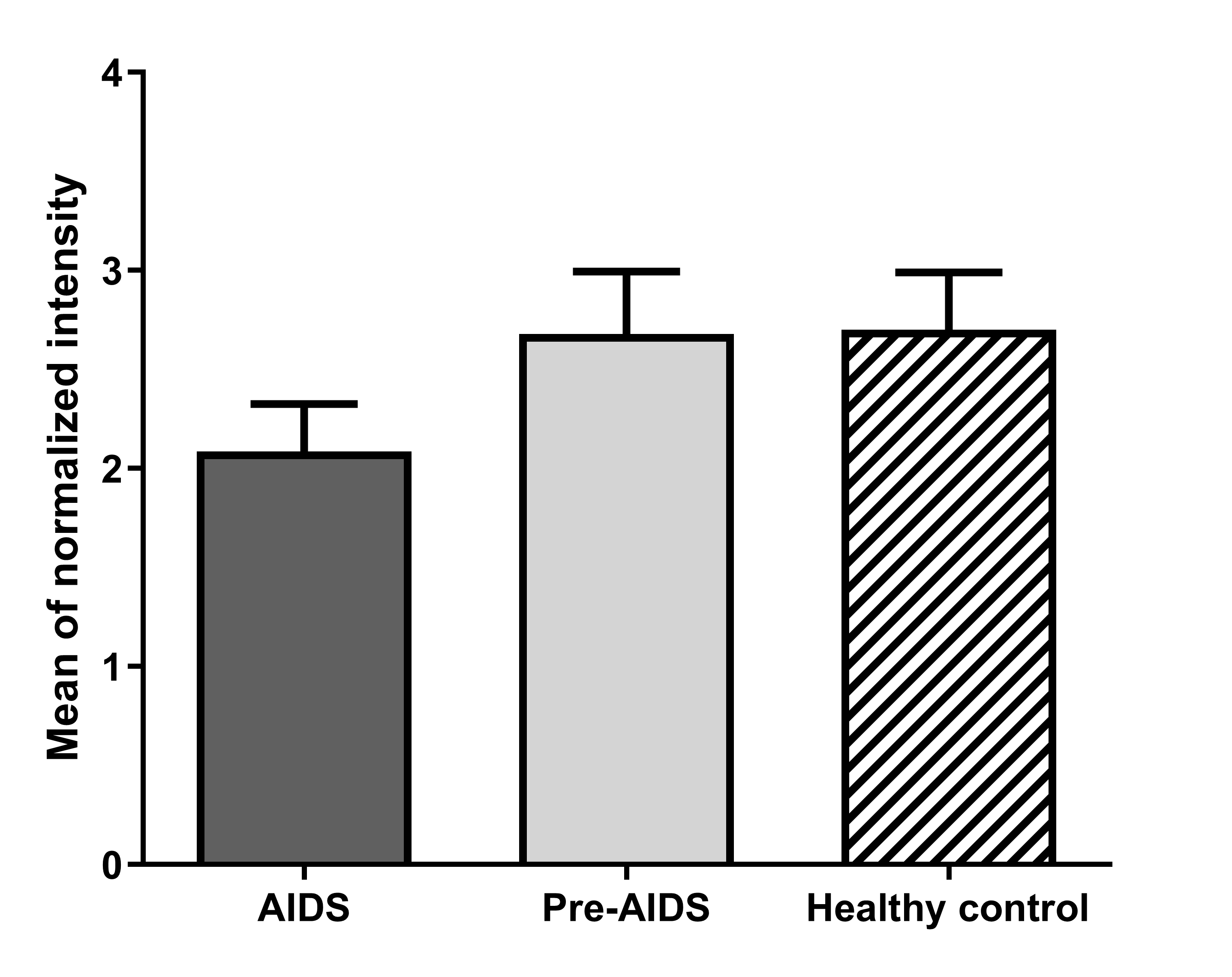

Supplement: Supplementary Figure 2 — Butyric acid at different stages of HIV infection. [file Image_2.tif]

Spearman Correlation Heatmap

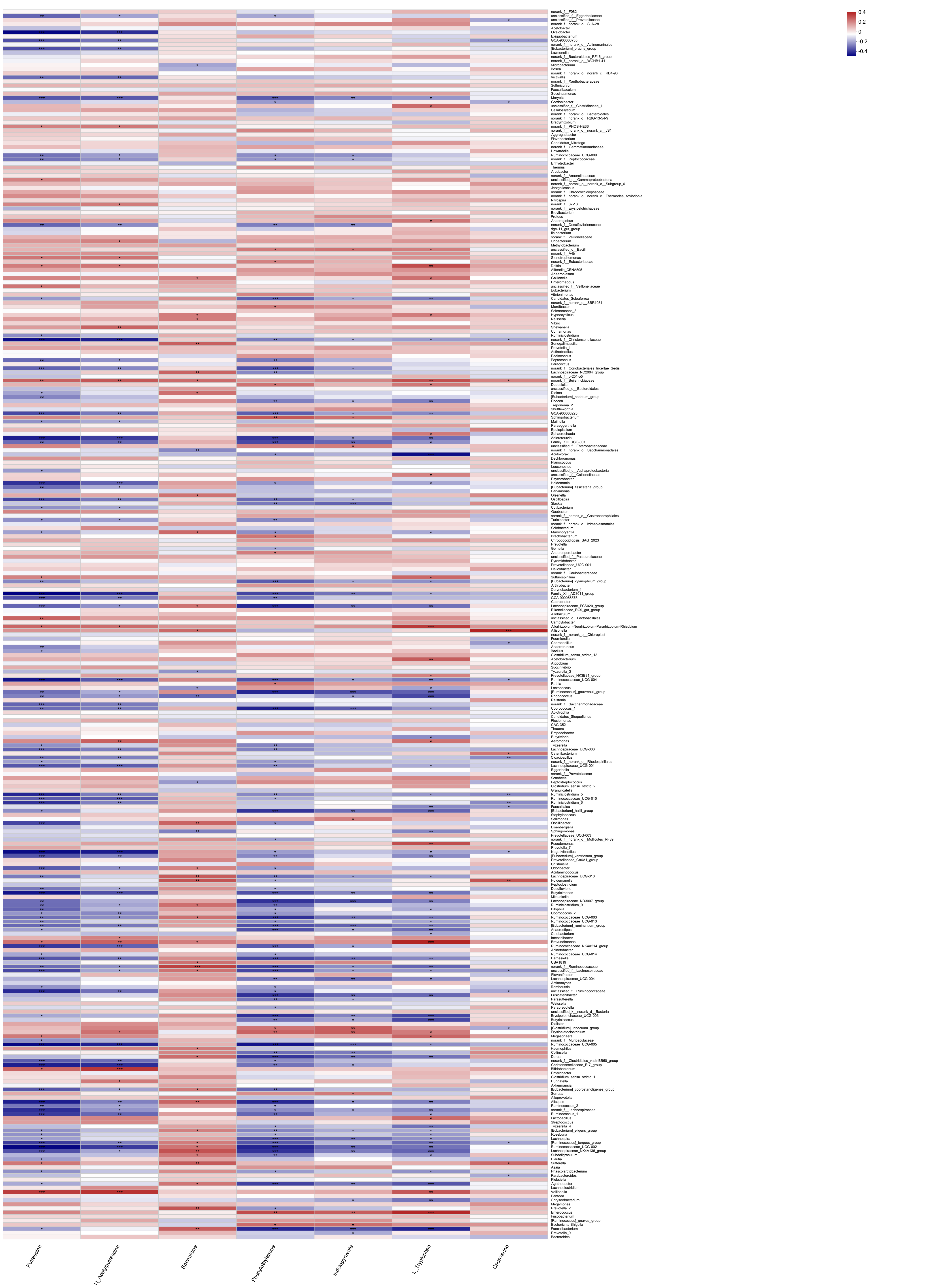

Supplement: Supplementary Figure 3 — The association between bacterial genera and metabolites positively correlated with immune activation and microbial translocation. [file DataSheet_1.pdf]
