# Supplementary material for: The altered metabolites contributed by dysbiosis of gut microbiota are associated with microbial translocation and immune activation during HIV infection
Source: Front Immunol. 2023 Jan 4;13:1020822. doi: 10.3389/fimmu.2022.1020822 (PMC9845923; doi:10.3389/fimmu.2022.1020822)
Supplement: Supplementary Figure 6 — The association between bacterial species and metabolites negatively correlated with immune activation and microbial translocation. [file DataSheet_4.pdf]

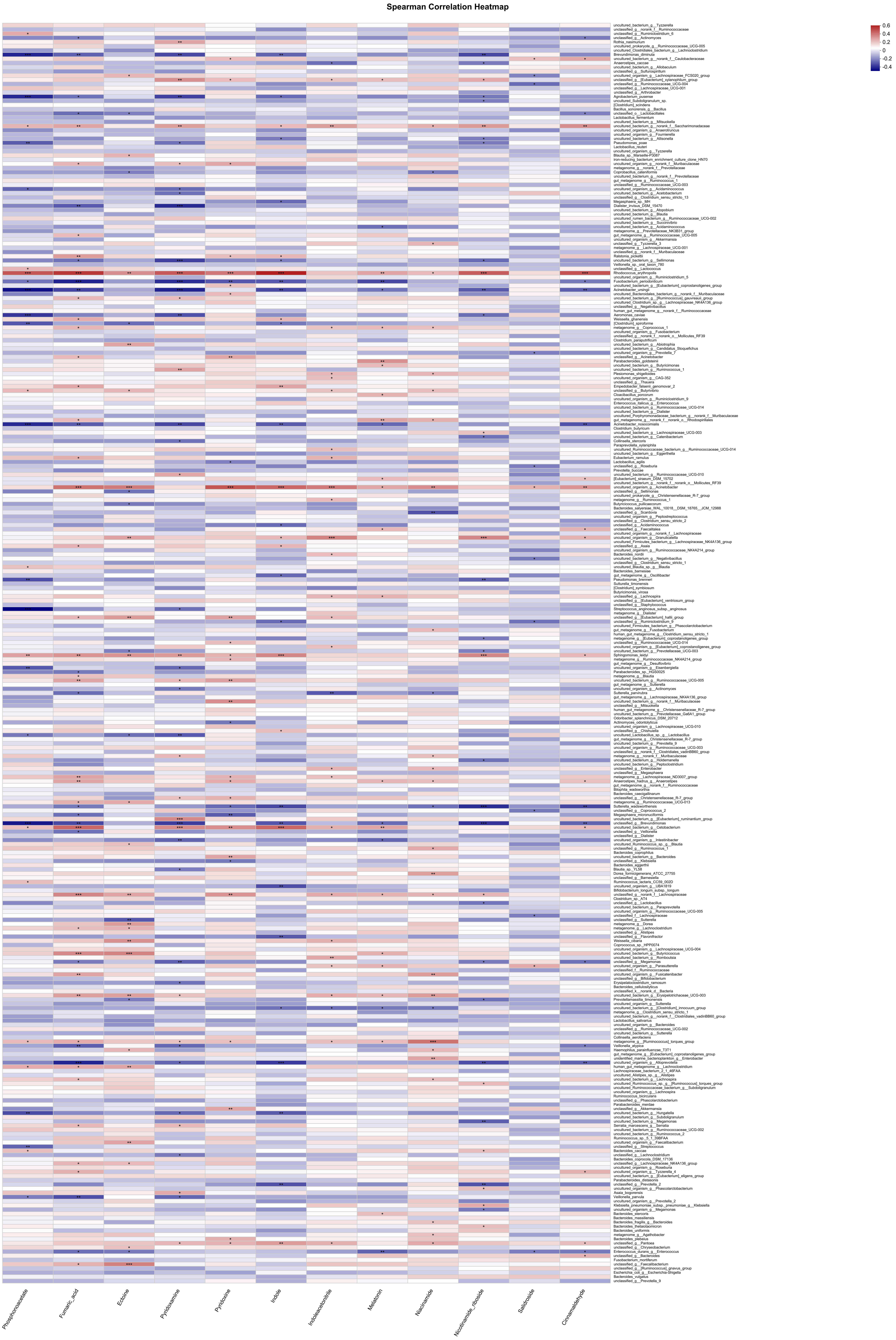
Spearman Correlation Heatmap
0.6
0.4
0.2
0
-0.2
-0.4
Phosphonoacetate
Fumaric_acid
Ectoine
Pyridoxamine
Pyridoxine
Indole
Indoleacetonitrile
Melatonin
Niacinamide
Nicotinamide_riboside
Salidroside
Cinnamaldehyde
