# Supplementary material for: The altered metabolites contributed by dysbiosis of gut microbiota are associated with microbial translocation and immune activation during HIV infection
Source: Front Immunol. 2023 Jan 4;13:1020822. doi: 10.3389/fimmu.2022.1020822 (PMC9845923; doi:10.3389/fimmu.2022.1020822)
Supplement: Supplementary file 7 [file Table_1.docx]

**Supplementary tables**

**Table S1**. Difference in markers of microbioal translocation and immune activation at different stages of HIV infection

|  | AIDS  (n=41) | Pre-AIDS  (n=39) | Healthy control  (n=34) | *P* value |
| --- | --- | --- | --- | --- |
| EndoCAb IgM (MMU/ml) |  |  |  |  |
| Median (IQR) | 27.63 (16.25, 38.78) | 61.79 (31.02, 87.87) | 49.45 (37.88, 74.32) | <0.001 |
| sCD14 (pg/ml) |  |  |  |  |
| Median (IQR) | 2.55×10^6^  (2.24×10^6^, 3.25×10^6^) | 2.14×10^6^  (1.54×10^6^ , 2.56×10^6^) | 1.71×10^6^  (1.46×10^6^, 2.03×10^6^) | <0.001 |
| The proportion of activated CD4+ T cells (%) |  |  |  |  |
| Median (IQR) | 23.59 (16.79, 25.06) | 5.15 (2.94, 9.87) | 1.23 (0.81, 1.78) | <0.001 |
| The proportion of activated CD8+ T cells (%) |  |  |  |  |
| Median (IQR) | 21.69 (16.05, 27.95) | 16.44 (8.08, 23.88) | 1.81 (1.20, 2.39) | <0.001 |

**Table S2**. Difference of gut microbiota among different stages of HIV infection

|  | AIDS (Mean, ‰) | Pre-AIDS (Mean, ‰) | Healthy control (Mean, ‰) | FDR *P* value |
| --- | --- | --- | --- | --- |
| Bacterial Genus |  |  |  |  |
| *Enterococcus* | 62.330 | 1.422 | 0.364 | <0.001 |
| *Brevundimonas* | 0.737 | 3.422 | 0.049 | <0.001 |
| *Aeromonas* | 0.265 | 0.784 | 0.075 | <0.001 |
| *Pseudomonas* | 0.754 | 1.626 | 0.116 | 0.006 |
| *Faecalibacterium* | 14.230 | 43.710 | 70.640 | <0.001 |
| *Lachnospira* | 2.409 | 9.330 | 14.80 | <0.001 |
| *Ruminococcaceae_UCG-002* | 3.558 | 10.430 | 12.91 | 0.001 |
| *Roseburia* | 6.260 | 7.746 | 11.48 | 0.003 |
| *Dorea* | 0.651 | 3.955 | 7.159 | <0.001 |
| *Prevotella_9* | 37.990 | 177.400 | 133.800 | 0.036 |
| *Fusicatenibacter* | 1.311 | 2.776 | 2.580 | 0.012 |
| *Lactobacillus* | 9.245 | 9.494 | 0.410 | <0.001 |
| Bacterial Species |  |  |  |  |
| *Enterococcus durans* | 61.320 | 1.336 | 0.072 | <0.001 |
| *Acinetobacter nosocomialis* | 0.762 | 0.325 | 0.001 | <0.001 |
| *Aeromonas caviae* | 0.213 | 0.704 | 0.017 | <0.001 |
| *Anaerostipes hadrus* | 0.694 | 0.806 | 2.078 | 0.015 |
| *Eubacterium ramulus* | 0.239 | 0.378 | 0.780 | 0.014 |
| *Bacteroides plebeius* | 3.827 | 27.775 | 21.540 | 0.046 |
| *Dorea formicigenerans* | 0.198 | 1.459 | 4.121 | <0.001 |

**Table S3.** Correlation of altered metabolites during HIV infection with microbioal translocation and immune activation

|  | EndoCAb IgM | |  | sCD14 | |  | Activated CD4+ T cells | |  | Activated CD8+ T cells | |
| --- | --- | --- | --- | --- | --- | --- | --- | --- | --- | --- | --- |
|  | r value | P value |  | r value | P value |  | r value | P value |  | r value | P value |
| Low-level metabolites in HIV-positive individuals |  |  |  |  |  |  |  |  |  |  |  |
| Phosphonoacetate | 0.149 | 0.138 |  | -0.191 | 0.053 |  | -0.317 | 0.004 |  | -0.456 | <0.001 |
| Pyridoxamine | -0.028 | 0.783 |  | -0.259 | 0.008 |  | -0.376 | 0.001 |  | -0.444 | <0.001 |
| Pyridoxine | -0.011 | 0.917 |  | -0.147 | 0.137 |  | -0.298 | 0.008 |  | -0.273 | 0.015 |
| Niacinamide | 0.161 | 0.110 |  | -0.283 | 0.004 |  | -0.280 | 0.012 |  | -0.342 | 0.002 |
| Nicotinamide riboside | -0.016 | 0.873 |  | -0.232 | 0.018 |  | -0.275 | 0.014 |  | -0.307 | 0.006 |
| Indole | -0.052 | 0.606 |  | -0.100 | 0.311 |  | -0.305 | 0.006 |  | -0.384 | <0.001 |
| 3-Indoleacetonitrile | 0.011 | 0.912 |  | -0.178 | 0.071 |  | -0.343 | 0.002 |  | -0.314 | 0.005 |
| Ectoine | 0.007 | 0.948 |  | -0.234 | 0.017 |  | -0.376 | 0.001 |  | -0.375 | 0.001 |
| Salidroside | 0.035 | 0.727 |  | -0.071 | 0.471 |  | -0.191 | 0.092 |  | -0.199 | 0.079 |
| Cinnamaldehyde | -0.057 | 0.573 |  | -0.283 | 0.004 |  | -0.388 | <0.001 |  | -0.402 | <0.001 |
| Melatonin | -0.036 | 0.720 |  | -0.164 | 0.096 |  | -0.405 | <0.001 |  | -0.471 | <0.001 |
| Fumaric acid | 0.035 | 0.727 |  | -0.339 | <0.001 |  | -0.438 | <0.001 |  | -0.461 | <0.001 |
| High-level metabolites in HIV-positive individuals |  |  |  |  |  |  |  |  |  |  |  |
| Putrescine | 0.030 | 0.765 |  | 0.092 | 0.351 |  | 0.232 | 0.040 |  | 0.325 | 0.003 |
| Cadaverine | 0.033 | 0.746 |  | 0.036 | 0.717 |  | -0.060 | 0.597 |  | 0.196 | 0.084 |
| N-Acetylputrescine | 0.053 | 0.602 |  | 0.101 | 0.307 |  | 0.152 | 0.181 |  | 0.259 | 0.021 |
| Phenylethylamine | -0.234 | 0.019 |  | 0.336 | <0.001 |  | 0.455 | <0.001 |  | 0.434 | <0.001 |
| Spermidine | 0.233 | 0.020 |  | -0.031 | 0.759 |  | 0.056 | 0.626 |  | 0.236 | 0.037 |
| Indolepyruvate | -0.204 | 0.042 |  | 0.205 | 0.037 |  | 0.368 | 0.001 |  | 0.361 | 0.001 |
| L-Tryptophan | -0.192 | 0.056 |  | 0.326 | 0.001 |  | 0.495 | <0.001 |  | 0.492 | <0.001 |

**Table S4.** Correlation of gut microbiota with tryptophan and its catabolites

| Gut microbiota | Indolepyruvate | |  | L-Tryptophan | |  | Indole | |  | 3-Indoleacetonitrile | |  |
| --- | --- | --- | --- | --- | --- | --- | --- | --- | --- | --- | --- | --- |
|  | r value | P value |  | r value | P value |  | r value | P value |  | r value | P value |  |
| Bacterial genus |  |  |  |  |  |  |  |  |  |  |  |  |
| *Enterococcus* | 0.253 | 0.007 |  | 0.357 | <0.001 |  | -0.056 | 0.559 |  | -0.137 | 0.147 |  |
| *Lactobacillus* | 0.072 | 0.448 |  | 0.232 | 0.013 |  | -0.021 | 0.829 |  | -0.119 | 0.208 |  |
| *Brevundimonas* | 0.151 | 0.110 |  | 0.363 | <0.001 |  | -0.263 | 0.005 |  | -0.042 | 0.659 |  |
| *Pseudomonas* | 0.046 | 0.628 |  | 0.284 | 0.002 |  | -0.008 | 0.937 |  | -0.021 | 0.829 |  |
| *Aeromonas* | 0.021 | 0.829 |  | 0.217 | 0.021 |  | -0.110 | 0.245 |  | 0.021 | 0.829 |  |
| *Prevotella-9* | -0.189 | 0.046 |  | -0.014 | 0.885 |  | -0.082 | 0.385 |  | 0.080 | 0.397 |  |
| *Faecalibacterium* | -0.329 | <0.001 |  | -0.409 | <0.001 |  | -0.060 | 0.527 |  | 0.177 | 0.061 |  |
| *Ruminococcaceae-UCG-002* | -0.304 | 0.001 |  | -0.282 | 0.002 |  | -0.018 | 0.846 |  | 0.128 | 0.176 |  |
| *Lachnospira* | -0.290 | 0.002 |  | -0.235 | 0.012 |  | 0.026 | 0.782 |  | 0.178 | 0.059 |  |
| *Roseburia* | -0.143 | 0.130 |  | -0.219 | 0.020 |  | -0.076 | 0.421 |  | 0.107 | 0.260 |  |
| *Dorea* | -0.290 | 0.002 |  | -0.279 | 0.003 |  | -0.049 | 0.606 |  | 0.126 | 0.183 |  |
| *Fusicatenibacter* | -0.298 | 0.001 |  | -0.302 | 0.001 |  | 0.039 | 0.678 |  | 0.136 | 0.150 |  |
| Bacterial species |  |  |  |  |  |  |  |  |  |  |  |  |
| *Enterococcus durans* | 0.222 | 0.018 |  | 0.369 | <0.001 |  | -0.068 | 0.473 |  | -0.131 | 0.166 |  |
| *Acinetobacter nosocomialis* | 0.183 | 0.052 |  | 0.337 | <0.001 |  | -0.258 | 0.006 |  | -0.091 | 0.340 |  |
| *Aeromonas caviae* | 0.065 | 0.494 |  | 0.291 | 0.002 |  | -0.165 | 0.081 |  | -0.026 | 0.782 |  |
| *Anaerostipes hadrus* | -0.222 | 0.018 |  | -0.267 | 0.004 |  | 0.147 | 0.120 |  | 0.082 | 0.385 |  |
| *Eubacterium ramulus* | -0.310 | 0.001 |  | -0.288 | 0.002 |  | 0.011 | 0.905 |  | 0.220 | 0.019 |  |
| *Bacteroides plebeius* | -0.105 | 0.267 |  | -0.064 | 0.504 |  | 0.046 | 0.629 |  | 0.088 | 0.357 |  |
| *Dorea formicigenerans* | -0.297 | 0.001 |  | -0.273 | 0.003 |  | -0.031 | 0.748 |  | 0.184 | 0.051 |  |

**Table S5.** Correlation of gut microbiota with anti-inflammatory metabolites

| Gut microbiota | Pyridoxamine | |  | Pyridoxine | |  | Niacinamide | |  | Nicotinamide riboside | |  | Ectoine | |  | Salidroside | |  | Cinnamaldehyde | |  | Melatonin | |  | Fumaric acid | |
| --- | --- | --- | --- | --- | --- | --- | --- | --- | --- | --- | --- | --- | --- | --- | --- | --- | --- | --- | --- | --- | --- | --- | --- | --- | --- | --- |
|  | r value | P value |  | r value | P value |  | r value | P value |  | r value | P value |  | r value | P value |  | r value | P value |  | r value | P value |  | r value | P value |  | r value | P value |
| Bacterial genus |  |  |  |  |  |  |  |  |  |  |  |  |  |  |  |  |  |  |  |  |  |  |  |  |  |  |
| *Enterococcus* | -0.058 | 0.543 |  | -0.044 | 0.643 |  | -0.150 | 0.112 |  | -0.087 | 0.362 |  | -0.193 | 0.040 |  | -0.182 | 0.053 |  | -0.184 | 0.051 |  | -0.227 | 0.016 |  | -0.195 | 0.038 |
| *Lactobacillus* | -0.261 | 0.005 |  | -0.025 | 0.789 |  | -0.183 | 0.053 |  | -0.245 | 0.009 |  | -0.156 | 0.100 |  | -0.042 | 0.657 |  | -0.048 | 0.614 |  | -0.136 | 0.150 |  | -0.055 | 0.561 |
| *Brevundimonas* | -0.337 | <0.001 |  | 0.037 | 0.701 |  | -0.021 | 0.821 |  | -0.346 | 0.000 |  | -0.074 | 0.438 |  | 0.037 | 0.695 |  | -0.233 | 0.013 |  | -0.214 | 0.023 |  | -0.284 | 0.002 |
| *Pseudomonas* | -0.157 | 0.098 |  | 0.194 | 0.040 |  | -0.107 | 0.261 |  | -0.264 | 0.005 |  | -0.068 | 0.474 |  | 0.055 | 0.563 |  | -0.073 | 0.444 |  | -0.085 | 0.372 |  | -0.058 | 0.543 |
| *Aeromonas* | -0.243 | 0.009 |  | 0.188 | 0.046 |  | 0.052 | 0.583 |  | -0.150 | 0.113 |  | -0.062 | 0.516 |  | 0.188 | 0.047 |  | -0.106 | 0.263 |  | -0.072 | 0.449 |  | -0.114 | 0.231 |
| *Prevotella_9* | -0.009 | 0.922 |  | 0.073 | 0.445 |  | 0.066 | 0.485 |  | -0.083 | 0.381 |  | -0.013 | 0.895 |  | -0.022 | 0.815 |  | -0.104 | 0.271 |  | 0.032 | 0.740 |  | -0.066 | 0.489 |
| *Faecalibacterium* | 0.021 | 0.822 |  | 0.108 | 0.257 |  | 0.131 | 0.165 |  | 0.016 | 0.867 |  | 0.330 | <0.001 |  | 0.038 | 0.688 |  | 0.060 | 0.527 |  | 0.164 | 0.083 |  | 0.211 | 0.025 |
| *Ruminococcaceae-UCG-002* | 0.108 | 0.256 |  | 0.117 | 0.215 |  | 0.147 | 0.121 |  | 0.090 | 0.342 |  | 0.116 | 0.221 |  | -0.092 | 0.334 |  | 0.022 | 0.816 |  | 0.115 | 0.226 |  | 0.142 | 0.132 |
| *Lachnospira* | -0.003 | 0.976 |  | 0.086 | 0.367 |  | 0.187 | 0.047 |  | 0.071 | 0.452 |  | 0.196 | 0.037 |  | 0.094 | 0.324 |  | 0.134 | 0.158 |  | 0.116 | 0.222 |  | 0.180 | 0.057 |
| *Roseburia* | -0.011 | 0.906 |  | 0.007 | 0.939 |  | 0.152 | 0.108 |  | 0.116 | 0.221 |  | 0.115 | 0.223 |  | -0.116 | 0.223 |  | 0.053 | 0.574 |  | 0.063 | 0.506 |  | 0.152 | 0.108 |
| *Dorea* | 0.078 | 0.409 |  | 0.125 | 0.186 |  | 0.210 | 0.026 |  | 0.065 | 0.494 |  | 0.239 | 0.011 |  | 0.010 | 0.913 |  | 0.064 | 0.498 |  | 0.032 | 0.734 |  | 0.146 | 0.123 |
| *Fusicatenibacter* | 0.050 | 0.602 |  | 0.178 | 0.059 |  | 0.263 | 0.005 |  | 0.030 | 0.751 |  | 0.146 | 0.123 |  | 0.032 | 0.739 |  | 0.072 | 0.448 |  | 0.155 | 0.101 |  | 0.245 | 0.009 |
| Bacterial species |  |  |  |  |  |  |  |  |  |  |  |  |  |  |  |  |  |  |  |  |  |  |  |  |  |  |
| *Enterococcus durans* | -0.069 | 0.467 |  | -0.044 | 0.646 |  | -0.148 | 0.117 |  | -0.083 | 0.383 |  | -0.201 | 0.033 |  | -0.195 | 0.039 |  | -0.202 | 0.032 |  | -0.242 | 0.010 |  | -0.219 | 0.020 |
| *Acinetobacter nosocomialis* | -0.294 | 0.002 |  | 0.007 | 0.945 |  | -0.044 | 0.644 |  | -0.147 | 0.120 |  | -0.136 | 0.150 |  | 0.031 | 0.743 |  | -0.272 | 0.004 |  | -0.234 | 0.012 |  | -0.255 | 0.006 |
| *Aeromonas caviae* | -0.280 | 0.003 |  | 0.138 | 0.144 |  | 0.006 | 0.946 |  | -0.223 | 0.018 |  | -0.101 | 0.288 |  | 0.142 | 0.132 |  | -0.159 | 0.094 |  | -0.118 | 0.212 |  | -0.183 | 0.052 |
| *Anaerostipes hadrus* | 0.084 | 0.374 |  | 0.231 | 0.014 |  | 0.192 | 0.042 |  | 0.142 | 0.133 |  | 0.170 | 0.072 |  | 0.059 | 0.533 |  | 0.189 | 0.045 |  | 0.210 | 0.026 |  | 0.244 | 0.009 |
| *Eubacterium ramulus* | 0.153 | 0.106 |  | 0.083 | 0.384 |  | 0.180 | 0.056 |  | 0.141 | 0.138 |  | 0.162 | 0.086 |  | -0.100 | 0.294 |  | 0.039 | 0.682 |  | 0.114 | 0.229 |  | 0.230 | 0.014 |
| *Bacteroides plebeius* | 0.026 | 0.782 |  | 0.224 | 0.017 |  | 0.180 | 0.056 |  | 0.041 | 0.664 |  | 0.021 | 0.828 |  | -0.007 | 0.938 |  | 0.016 | 0.870 |  | 0.080 | 0.401 |  | 0.124 | 0.189 |
| *Dorea formicigenerans* | -0.002 | 0.985 |  | 0.125 | 0.188 |  | 0.259 | 0.006 |  | 0.083 | 0.380 |  | 0.142 | 0.133 |  | -0.031 | 0.742 |  | -0.011 | 0.910 |  | 0.047 | 0.621 |  | 0.142 | 0.133 |

**Table S6.** Correlation of gut microbiota with biogenic amines and phosphonoacetate

| Gut microbiota | Putrescine | |  | Cadaverine | |  | Phenylethylamine | |  | N-Acetylputrescine | |  | Spermidine | |  | Phosphonoacetate | |
| --- | --- | --- | --- | --- | --- | --- | --- | --- | --- | --- | --- | --- | --- | --- | --- | --- | --- |
|  | r value | P value |  | r value | P value |  | r value | P value |  | r value | P value |  | r value | P value |  | r value | P value |
| Bacterial genus |  |  |  |  |  |  |  |  |  |  |  |  |  |  |  |  |  |
| *Enterococcus* | 0.182 | 0.054 |  | 0.057 | 0.546 |  | 0.288 | 0.002 |  | 0.088 | 0.356 |  | -0.015 | 0.873 |  | -0.106 | 0.262 |
| *Lactobacillus* | 0.131 | 0.166 |  | 0.119 | 0.208 |  | 0.070 | 0.464 |  | 0.178 | 0.060 |  | 0.015 | 0.871 |  | -0.231 | 0.014 |
| *Brevundimonas* | 0.218 | 0.020 |  | 0.146 | 0.122 |  | 0.121 | 0.199 |  | 0.247 | 0.008 |  | 0.195 | 0.038 |  | -0.383 | <0.001 |
| *Pseudomonas* | 0.070 | 0.461 |  | 0.063 | 0.511 |  | 0.099 | 0.299 |  | 0.129 | 0.175 |  | 0.084 | 0.374 |  | -0.289 | 0.002 |
| *Aeromonas* | 0.170 | 0.071 |  | 0.029 | 0.762 |  | -0.046 | 0.632 |  | 0.249 | 0.007 |  | 0.179 | 0.058 |  | -0.279 | 0.003 |
| *Prevotella-9* | -0.031 | 0.743 |  | 0.095 | 0.318 |  | -0.131 | 0.167 |  | -0.052 | 0.586 |  | 0.084 | 0.377 |  | -0.079 | 0.404 |
| *Faecalibacterium* | -0.203 | 0.031 |  | 0.033 | 0.733 |  | -0.406 | <0.001 |  | -0.077 | 0.420 |  | 0.255 | 0.006 |  | 0.148 | 0.119 |
| *Ruminococcaceae-UCG-002* | -0.463 | <0.001 |  | -0.058 | 0.539 |  | -0.410 | <0.001 |  | -0.375 | <0.001 |  | 0.212 | 0.024 |  | 0.133 | 0.162 |
| *Lachnospira* | -0.209 | 0.026 |  | -0.120 | 0.207 |  | -0.335 | <0.001 |  | -0.106 | 0.262 |  | 0.124 | 0.192 |  | 0.112 | 0.238 |
| *Roseburia* | -0.231 | 0.014 |  | -0.006 | 0.948 |  | -0.196 | 0.037 |  | -0.124 | 0.191 |  | 0.088 | 0.354 |  | 0.064 | 0.502 |
| *Dorea* | -0.169 | 0.073 |  | 0.125 | 0.187 |  | -0.382 | <0.001 |  | -0.092 | 0.335 |  | 0.225 | 0.017 |  | 0.087 | 0.362 |
| *Fusicatenibacter* | -0.174 | 0.065 |  | 0.055 | 0.564 |  | -0.390 | <0.001 |  | -0.118 | 0.212 |  | 0.168 | 0.075 |  | 0.120 | 0.207 |
| Bacterial species |  |  |  |  |  |  |  |  |  |  |  |  |  |  |  |  |  |
| *Enterococcus durans* | 0.171 | 0.070 |  | 0.054 | 0.566 |  | 0.254 | 0.007 |  | 0.072 | 0.449 |  | -0.005 | 0.955 |  | -0.102 | 0.283 |
| *Acinetobacter nosocomialis* | 0.220 | 0.019 |  | 0.078 | 0.411 |  | 0.106 | 0.262 |  | 0.246 | 0.009 |  | 0.215 | 0.022 |  | -0.351 | <0.001 |
| *Aeromonas caviae* | 0.232 | 0.013 |  | 0.070 | 0.461 |  | 0.031 | 0.744 |  | 0.283 | 0.002 |  | 0.209 | 0.026 |  | -0.326 | <0.001 |
| *Anaerostipes hadrus* | -0.230 | 0.014 |  | 0.002 | 0.983 |  | -0.307 | <0.001 |  | -0.161 | 0.088 |  | -0.013 | 0.889 |  | 0.075 | 0.430 |
| *Eubacterium ramulus* | -0.317 | <0.001 |  | -0.052 | 0.583 |  | -0.380 | <0.001 |  | -0.286 | 0.002 |  | 0.017 | 0.855 |  | 0.182 | 0.053 |
| *Bacteroides plebeius* | -0.057 | 0.549 |  | -0.032 | 0.737 |  | -0.189 | 0.045 |  | -0.031 | 0.744 |  | 0.143 | 0.130 |  | 0.027 | 0.775 |
| *Dorea formicigenerans* | -0.130 | 0.170 |  | 0.110 | 0.245 |  | -0.344 | <0.001 |  | -0.066 | 0.488 |  | 0.207 | 0.028 |  | 0.082 | 0.390 |
