# Supplementary material for: The altered metabolites contributed by dysbiosis of gut microbiota are associated with microbial translocation and immune activation during HIV infection
Source: Front Immunol. 2023 Jan 4;13:1020822. doi: 10.3389/fimmu.2022.1020822 (PMC9845923; doi:10.3389/fimmu.2022.1020822)

Spearman Correlation Heatmap

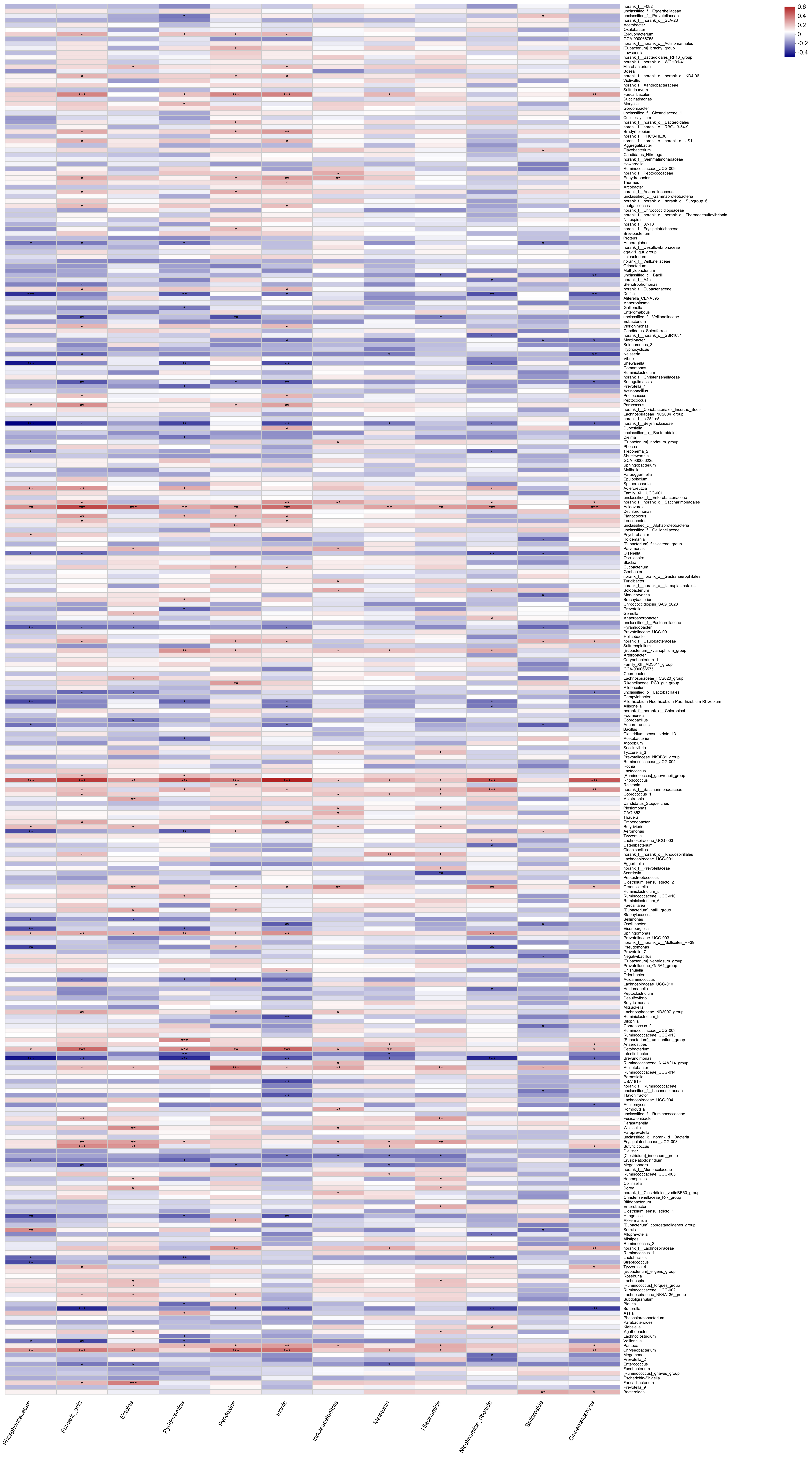

Supplement: Supplementary Figure 5 — The association between bacterial genera and metabolites negatively correlated with immune activation and microbial translocation. [file DataSheet_3.pdf]
